# Factors Related to Early Recurrence of Idiopathic Clubfoot Post the Ponseti Method

**DOI:** 10.5704/MOJ.1911.005

**Published:** 2019-11

**Authors:** N Limpaphayom, P Sailohit

**Affiliations:** Department of Orthopaedics, Chulalongkorn University, Bangkok, Thailand; * Department of Orthopaedics, Police General Hospital, Bangkok, Thailand

**Keywords:** braces, compliance, clubfoot, recurrence, treatment

## Abstract

**Introduction:** Idiopathic clubfoot or congenital talipes equinovarus (CTEV) is managed by the Ponseti method worldwide; however, the recurrence of the deformity is a challenging problem. The purpose was to review the factors associated with early recurrence of CTEV post the Ponseti method.

**Materials and Methods:** During 2011-2016, 34 infants with 52 CTEV, who underwent the Ponseti method and a minimum follow-up period of six months, were reviewed. Twenty-two infants (65%) were male, and 18 infants (53%) had bilateral CTEV. Recurrence of CTEV was defined as a reappearance of at least one of the four components of the deformity. The association between recurrence and factors, including age, gender, bilaterality, family geography, type of principal caregiver, severity at presentation, centre where the Ponseti method was initiated, compliance to foot abduction brace (FAB), practice of stretching exercise, type of FAB, and complications of casting, were evaluated using univariate logistic regression analysis.

**Results:** The median age at initiation of the treatment was 3.4 (IQR; 2.1-12.6) weeks. A median of six (range; 3-12) casts were required. Tenotomy was performed in 32/34 (94%) of cases. Recurrence occurred in 14/52 feet (27%) at an average follow-up period of 2.3±1.1 years. Non-compliance to FAB protocol began at an average age of 11.2±6.5 months, and significantly increased the risk of recurrence during the weaning phase [OR (95%CI)=8.4 (1.2-92.4), p=0.03]. Other factors were not associated with the recurrence.

**Conclusion:** Non-compliance to FAB occurred early during the treatment and related to a risk of recurrence of CTEV. Physicians should encourage the parents and/or guardians to follow the protocol to decrease the risk of recurrence.

## Introduction

Idiopathic clubfoot or congenital talipes equinovarus (CTEV) is a well-known complex, congenital foot deformity composed of four components, namely: equinus, hindfoot varus, forefoot adduction and cavus^1^. Conservative treatment by manipulating and casting utilised the Ponseti method is the mainstay of practice and satisfactory results have been achieved by several authors^2-6^. The use of a foot abduction brace (FAB) after initial correction is mandatory to maintain the correction and to prevent the recurrence of CTEV^1, 6-9^. However, this is a challenging process, which requires cooperation from the parents or guardians^1, 3, 10, 11^.

The recurrence after initial correction of CTEV leads to further treatment and untoward outcomes. These include more casts, repeated tenotomy, and rarely more extensive surgical procedures which could affect the function of the patients in the long run^1^. Factors associated with the recurrence of CTEV could be the patient, physician, or caregiver-associated characteristics^1, 12-15^. Non-modifiable factors include high grade of deformity, ethnicity, and parental educational level^1, 6, 12, 16^. An important modifiable factor is non-compliance to FAB or practice of stretching exercise^1, 6, 9, 15, 16^. Physicians managing patients with CTEV should be aware of these factors before initiating the Ponseti method.

At our institution, the selective soft tissue release procedure^17^ become less favorable as we adopt the Ponseti method as a standard treatment for CTEV since 2011. The purpose of this study was to investigate clinical factors related to the recurrence of CTEV after the Ponseti method.

## Materials and Methods

We retrospectively reviewed 34 medical records [22 males (65%), 12 females (35%)] from 2011 to 2016 of children who were treated for idiopathic CTEV at our institution with a minimum follow-up period of six months after the completion of the casting process. Eighteen patients (53%) had bilateral CTEV while 16 patients (37%) had unilateral CTEV. Clubfeet with neuromuscular or syndromic in origin were excluded.

All patients underwent the Ponseti method, including a weekly foot manipulation and serial casting followed by percutaneous tendoachilles tenotomy (pTAL) if indicated. The procedure was performed by one physician (N.L.). To maintain the correction, FAB was used as previously described^1, 4^. Types of FAB utilised in the study were a fixed (Denis Browne bar) type and an articulated (Dobbs bar) type^4, 18^. The selection of FAB type was at parental discretion. Nineteen fixed type bars and 15 articulated type bars were used. The standard FAB protocol was administered regardless of bar type. The FAB was worn full time (23 hours) in the initial phase for the first three months followed by 18-23 hours during the weaning phase for another three months. Then the FAB was worn for 12-18 hours a day during the maintenance phase until the patient was four years old^8^.

Parents or guardians were asked to perform the stretching exercises for the patients, which included a passive range of motion of the involved ankle and foot at least 20 minutes twice a day. A squatting exercise was recommended in addition to the ankle stretching exercise after the walking age. The patients were followed at an outpatient clinic at a regular interval. Recurrence of CTEV was defined as a reappearance of at least one of the four components of CTEV^11, 19^.

Patient information extracted from medical record review included age at initiation of the treatment, gender, bilaterality, family geography, type of principal caregiver, and severity of CTEV at presentation. The severity of the deformity was graded from 1-4 according to the system of Dimeglio *et al*^20^. The patients were divided into two groups: grades 1-2 (mild to moderate) and grades 3-4 (severe to very severe). Treatment information included centre where the Ponseti method was initiated, compliance to FAB, compliance to the practice of stretching exercise, type of FAB, and complications of casting. Compliance to the use of FAB and stretching exercise were recorded in hours per day according to caregivers’ self-report. Non-compliance to FAB was defined as the duration of FAB use of lesser than 23 hours, 18-23 hours, and 12-18 hours in the initial phase, weaning phase, and maintenance phase, respectively^8^. Outcomes of interest were recurrence of CTEV, the duration between the initial correction and the detection of recurrence, and the need for recasting.

Data were reported as number and percent, or mean±standard deviation (SD) when appropriate. A case-control odds-ratio calculation was performed to evaluate parameters modelling for recurrence. Unadjusted odd ratios and 95% confidence interval (CI) were calculated. P-value of less than 0.05 was considered statistically significant. A post-hoc power analysis for a two-sample proportion was conducted. Statistical analysis was performed using Stata 13 (Stata Corp LP, College Station, TX, USA.). This study was approved by an ethical review board of the Faculty of Medicine, Chulalongkorn University, Thailand, COA 939/2017, and complied with the declaration of Helsinki 1975, as revised in 1983. For a retrospective study, formal inform consent was exempted.

## Results

The median age at initiation of the treatment was 3.4 (IQR; 2.1-12.6) with of 0.4 to 58.1 weeks. Six (18%) patients had Dimeglio grade 2 (moderate) and 28 (82%) patients had Dimeglio grades 3-4 (severe to very severe). The Ponseti method was initiated at our centre in 26 (76%) patients. A median of six (range; 3-12) casts were applied and pTAL was performed in an outpatient setting in 32/34 (94%) cases. All CTEV were clinically, fully corrected, defined as having a dorsiflexion at the ankle of 15º and an abduction of the forefoot at 60º-70º after completion of the casting.

At an average follow-up period of 2.3±1.1 (range; 0.6-4.2) years, 14/52 (27%) feet in 11/34 (32%) patients had recurrence at an average age of 26.4±11.9 months. There were recurrent equinus in five feet and recurrent metatarsus adductus in nine feet. Non-compliance to the FAB protocol began at an average age of 11.2±6.5 months. Odds ratio of patient- and treatment-associated factors related to the recurrence of CTEV are shown in Table I and II, respectively. Non-compliance to FAB during the weaning phase was significantly associated with recurrence with an odds ratio of 8.4 [95% CI; (1.2-92.4), p=0.03]. Age of initiation of the treatment did not influence the recurrence. Non-parental caregivers, CTEV with Dimeglio grade 3-4, non-compliance to FAB during the initial and maintenance phases, lack of practice of stretching exercise and the use of fixed type bar also demonstrated higher but non-significant odds of higher chance of recurrence. No association was found between other clinical factors and the recurrence of CTEV. A post-hoc chi-square power analysis for a two-sample proportion had a power of 87.3%.

**Table I:** Patients associated factors related to recurrence of idiopathic congenital talipes equinovarus post the Ponseti method

Factors		Recurrence	No Recurrence	OR (95%CI)	P
Number of patients		11 (32%)	23 (68%)		
Number of CTEV		14 (27%)	38 (73%)		
Age at initiation of treatment	> 12 weeks	3 (33%)	6 (67%)	1.1 (0.1-6.7)	0.62
	< 12 weeks	8 (32%)	17 (68%)		
Gender	Female	4 (33%)	8 (67%)	1.1 (0.2-5.9)	0.61
	Male	7 (32%)	15 (68%)		
Bilaterality	Bilateral	3 (17%)	15 (83%)	0.2 (0.03-1.19)	0.07
	Unilateral	8 (50%)	8 (50%)		
Family geography	Outside	7 (41%)	10 (59%)	2.3 (0.4-13.5)	0.23
	Bangkok metropolitan area	4 (23%)	13 (77%)		
Type of principal caregiver	Non-parents	2 (40%)	3 (60%)	1.5 (0.1-15.3)	0.53
	Parents	9 (31%)	20 (69%)		
Severity at presentation	Dimeglio 3-4 (feet)	13 (30%)	30 (70%)	3.5 (0.4-165.4)	0.23
	Dimeglio 2 (feet)	1 (11%)	8 (89%)		

**Table II:** Treatments associated factors related to recurrence of idiopathic congenital talipes equinovarus post the Ponseti method

Factors		Recurrence	No Recurrence	OR (95%CI)	P
Number of patients		11 (32%)	23 (68%)		
Number of CTEV		14 (27%)	38 (73%)		
Centre location	Outside facility	2 (25%)	6 (75%)	0.6 (0.1-4.6)	0.48
	KCMH	9 (35%)	17 (65%)		
Compliance to FAB -initial phase	No (<23 h/day)	5 (50%)	5 (50%)	3.0 (0.5-18.2)	0.16
	Yes (23 h/day)	6 (25%)	18 (75%)		
Wean phase	No (<12 h/day)	9 (53%)	8 (47%)	8.4 (1.2-92.4)	0.03
	Yes (12-18 h/day)	2 (11%)	15 (89%)		
Maintenance phase†	No (<12 h/day)	11 (40%)	16 (60%)	10.5 (0.5-201.8)	0.08
	Yes (12-18 h/day)	0	7 (100%)		
Regular practice of	No	3 (75%)	1 (25%)	8.3 (0.5-452.1)	0.09
stretching exercise	Yes	8 (27%)	22 (73%)		
Type of FAB	Fix (DB bar)	8 (42%)	11 (58%)	2.9 (0.5-20.8)	0.27
	Articulated joint (Dobbs bar)	3 (20%)	12 (80%)		
Complications of casting	Yes (feet)	5 (42%)	7 (58%)	2.5 (0.5-11.6)	0.19
	No (feet)	9 (23%)	31 (77%)		
Need for recasting	Yes (feet)	3 (33%)	8 (77%)	1.0 (0.1-5.3)	0.62
	No (feet)	11 (25%)	30 (75%)		

Data were presented as number of patient or feet (percent) as appropriate.

CI; confidence interval, CTEV; congenital talipes equinovarus, DB; Denis Browne, FAB; foot abduction brace, h; hours, KCMH; King

Chulalongkorn Memorial Hospital, OR; odds ratio

†Case-control odds ratio uses a 0.5 correction in the cell that contains a zero.

Ponseti casting was repeated in 11 patients (14 feet) with recurrent CTEV. Six feet responded to a repeat casting. A repeat pTAL was required in five feet with recurrent equinus, however, the plantigrade feet were achieved in 2/5 feet. Limited posterior and plantar release procedures were performed on one foot. The caregivers of the remaining patient (two feet) refused further treatment due to their personal beliefs. Recurrent metatarsus adductus was flexible and correctable. The caregivers of the remaining patients (three feet) preferred stretching exercises instead of casting. This group of patients was closely monitored for a fixed deformity or a possible dynamic supination of the forefoot during the gait. An additional procedure, e.g. tibialis anterior tendon transfer, was not required during the follow-up period.

## Discussion

An excellent outcome of CTEV managed by the Ponseti method is well established. An initial correction has been achieved in the majority of the cases^1, 12, 16^ and it is confirmed by our experience in this study. However, recurrence of the deformity after correction does occur with a recurrent rate between 14-41% being reported^6, 9^. In this retrospective review, non-compliance to the use of FAB to maintain CTEV correction was associated with a significant odds ratio of recurrence.

Bracing is an essential part of the Ponseti method. Standard protocol has been well outlined; however, many investigators note that bracing is a challenging process of the Ponseti method^3^. Previous studies have reported non-compliance rates to be 32-61%^9, 19^. Our study also had similar non-compliance rates. Dobbs *et al* used the criteria of non-compliance as complete discontinuation of the use of orthosis, which occurred during the first three months of bracing.

The parents or guardians often cited inconvenience as the main cause for not using the brace^1, 21^. Moreover, the FAB should be worn until at least four years of age and early discontinuation of FAB was another known factor for recurrence^22^. Non-compliance to the FAB protocol occurred at approximately one year after initiating treatment. In our experience, this coincided with the period when the parents had to go back to work after the parental-leave period and had to transfer the care of the patient to other people including grandparents and day care personnel. To improve the parents’ or guardians’ awareness, standardised information explaining the Ponseti method and the importance of compliance to FAB should be emphasised to the parents or guardians at the start of treatment^9, 19^. Regular discussion and support during each clinic visit and a follow-up phone call are very useful^9, 12^.

Other factors that demonstrated higher but not significant odds ratio of recurrence include CTEV with Dimeglio grades 3-4 at presentation, not performing the stretching exercise, the use of fix bar and the principal caregiver of the patient was non-parental, e.g. grandparents, family-helpers or day care personnel. Age at initiation of the treatment did not associate with the recurrence of the deformity. This finding is consistent with the recent report by Awang *et al*^23^. Previous studies used the Dimeglio classification to quantify the severity of the deformities^11, 20, 24^. The Dimeglio grade 3 (severe) and grade 4 (very severe) may be associated with a higher chance of recurrence^11, 25^. Dobbs *et al* reported that CTEV with Dimeglio grade 4 had an 8-fold chance of recurrence but this was not statistically significant^1^. Furthermore, Panjavi *et al* recently confirmed the association between severity of CTEV as rated by the Dimeglio classification and risk of recurrence^11^. This should remind the physician when applying the Ponseti method to this particular group of patients. On the other hand, Zhao *et al* devised a ratio of correction improvement using the Pirani scoring system and linked that to the risk of recurrence^6^.

Kite cited common errors of treatment that physicians relied on “Brace Maker” and “family exercise”^13^. The Ponseti method recommended stretching exercise performed by the parents or guardians as part of the daily care^1, 11^. We currently focus on routine stretching and squatting exercises to be performed when the patient has reached the walking age. We anticipate that this strategy may lessen the chance of recurrence as shown in previous reports^2, 11^. A recent study demonstrated that routine stretching exercise of the foot and ankle could be an important part of maintaining range of ankle and subtalar motions after a soft tissue release procedure for CTEV^17^. Another factor that can affect the recurrence is the type of FAB used^18^. Non-compliance to the FAB will definitely increase the recurrence rate of CTEV. We observed comparable odds of recurrence in the group of patients with fixed type bar and articulated type bar. The Denis Browne bar was preferred during the Ponseti era^4^ and it is still the primary choice of FAB in many centres^11, 26^. Our findings could be explained by the recent study by Agarwal *et al* who reported dynamic change of foot abduction measurements and successful outcomes utilising fixed FAB-Steenbeek type^7^. On the other hand, Chen *et al* reported a higher parental compliance rate when the articulated bar was first introduced^27^ and subsequently confirmed by Garg *et al* in a dynamic FAB model^18^. Application of foot pieces and the bar connector to a patient is easier when using the articulated type bar. The articulated type bar allows the knees and hips movements and could lower the blister formation^18, 27^. The major obstacle in our setting is the cost of commercial articulated bar as experienced by others^2, 3, 9, 21^. Before the treatment, the advantages and disadvantages of each type of FAB should be discussed with the parents or guardians. Janicki *et al* emphasised that the FAB should not be replaced by an ankle foot orthosis. The use of ankle foot orthoses demonstrated a notable recurrence rate when compared to the use of FAB^26^.

Family or domestic helper is common in our society, and corroborated by other studies conducted in different cultural background^10, 28^, and could dominate parental decision^2^. In contrast, Ramirez *et al* did not find any correlations between the parents’ or guardians’ factors and compliance rate^15^. According to our experience, it may be helpful to include other family members (e.g. grandparents) into the treatment scheme. This supports a recent work by Malagelada *et al* that bracing period could affect the entire family and recommended a supporting system to lower the burden^28^.

Despite the best effort, recurrences of CTEV do occur. Encouraging the parents or guardians and caregivers to adhere to the FAB protocol could lower the chance of recurrences^29^. A daily physiotherapy program adjunct to the Ponseti method shows an improvement of the Dimeglio scores^30^. Recurrent metatarsus adductus is prevalent in our study, corroborated by results from other studies^31, 32^. Expectant treatment could be employed in flexible deformities. A repeat episode of Ponseti casting was successfully utilised^4, 33^. Transfer of tibialis anterior tendon to lateral column of the foot improves long-term functions when dynamic forefoot supination observed during gait is evident^34^. The range of ankle dorsiflexion is related to the outcomes thus recurrent ankle equinus needs more aggressive management^35^. If recurrent heel varus combined with ankle dorsiflexion of less than 15º is detected, we prefer repeat casting and pTAL as advocated by Dobbs^33^. Marquez *et al* recommended a repeat Ponseti casting followed by a short leg walking cast until the range of ankle dorsiflexion of 20º was attained^35^. A soft tissue release procedure may be inevitable. It should follow an “a la carte” concept and progressively correct the remaining deformities and avoid dissection into the subtalar complex as possible^17, 33^. Several types of osteotomy around the midfoot have been proposed if the deformities become fixed^32^.

A few limitations need to be addressed. First, the mean 2.3 years of follow-up of the study is shorter than the recommended 4-year duration of FAB^1, 4, 8^. Moreover, due to the nature of the retrospective study, compliance to the treatment relied on the parents’ or guardians’ report. The prevalence of non-compliance to FAB could be higher in a longer-term follow-up. More recurrent CTEV could develop approaching the end of the FAB period and additional surgical procedures are a possibility. Events occurred in some parameters studied were not significant which may be due to the small sample size. Recruiting more CTEV patients would give an insight appraisal of other risk factors.

## Conclusion

In conclusion, non-compliance to FAB is related to the risk of CTEV recurrence after the Ponseti method. Family education and support are pivotal and physicians’ awareness is equally important. The parents and guardians should be encouraged to comply with the Ponseti method to decrease the risk of recurrence.
